# *N* ^2^,*N* ^2′^-Bis[4-(dimethyl­amino)­benzyl­idene]pyridine-2,6-dicarbohydrazide monohydrate

**DOI:** 10.1107/S1600536810038778

**Published:** 2010-10-02

**Authors:** Yong Wang, Yan Liang Sun

**Affiliations:** a Department of Chemistry, Liaocheng University, Liaocheng 252059, People’s Republic of China

## Abstract

In the title compound, C_25_H_27_N_7_O_2_·H_2_O, the bis­[4-(dimethyl­amino)­benzyl­idene]pyridine-2,6-dicarbohydrazide mol­ecule and the water mol­ecule are located on a twofold rotation axis. The benzene and pyridine rings form a dihedral angle of 17.13 (7)°. In the crystal, inter­molecular N—H⋯O and O—H⋯O hydrogen bonds link the mol­ecules into a two-dimensional supermolecular structure parallel to the *ab* plane.

## Related literature

For related structures, see: Cheng *et al.* (2007 ▶); Cheng & Liu (2008 ▶); Jia, Hu *et al.* (2006 ▶); Jia, Shi *et al.* (2006 ▶).
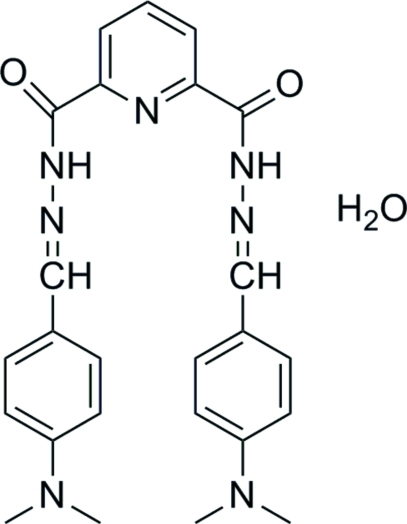


## Experimental

### 

#### Crystal data

C_25_H_27_N_7_O_2_·H_2_O
*M* *_r_* = 475.54
Monoclinic, 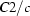

*a* = 8.5718 (11) Å
*b* = 10.2802 (14) Å
*c* = 27.112 (3) Å
β = 97.865 (1)°
*V* = 2366.7 (5) Å^3^
*Z* = 4
Mo *K*α radiation
μ = 0.09 mm^−1^
*T* = 298 K
0.36 × 0.31 × 0.16 mm

#### Data collection

Bruker SMART APEX CCD area-detector diffractometer
Absorption correction: multi-scan (*SADABS*; Sheldrick, 1996 ▶) *T*_min_ = 0.968, *T*_max_ = 0.986
5774 measured reflections
2076 independent reflections
1348 reflections with *I* > 2σ(*I*)
*R*_int_ = 0.039

#### Refinement

*R*[*F* ^2^ > 2σ(*F* ^2^)] = 0.062
*wR*(*F* ^2^) = 0.204
*S* = 1.05
2076 reflections
163 parameters
1 restraint
H atoms treated by a mixture of independent and constrained refinement
Δρ_max_ = 0.17 e Å^−3^
Δρ_min_ = −0.59 e Å^−3^

### 

Data collection: *SMART* (Siemens, 1996 ▶); cell refinement: *SAINT* (Siemens, 1996 ▶); data reduction: *SAINT*; program(s) used to solve structure: *SHELXS97* (Sheldrick, 2008 ▶); program(s) used to refine structure: *SHELXL97* (Sheldrick, 2008 ▶); molecular graphics: *SHELXTL* (Sheldrick, 2008 ▶); software used to prepare material for publication: *SHELXTL*.

## Supplementary Material

Crystal structure: contains datablocks I, global. DOI: 10.1107/S1600536810038778/rz2479sup1.cif

Structure factors: contains datablocks I. DOI: 10.1107/S1600536810038778/rz2479Isup2.hkl

Additional supplementary materials:  crystallographic information; 3D view; checkCIF report

## Figures and Tables

**Table 1:** Hydrogen-bond geometry (Å, °)

*D*—H⋯*A*	*D*—H	H⋯*A*	*D*⋯*A*	*D*—H⋯*A*
N2—H2*A*⋯O2^i^	0.86	2.23	2.967 (4)	143
O2—H2⋯O1^ii^	0.85 (3)	2.04 (4)	2.844 (3)	157 (3)

Symmetry codes: (i) 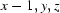; (ii) 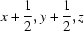.

**Fig. 1.**
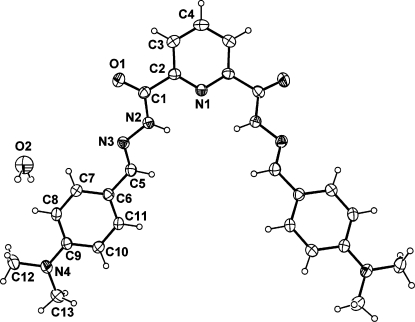
The molecular structure of the title compound, showing the atomic numbering scheme and 30% probability displacement ellipsoids. Unlabelled atoms are related to labelled atoms by (-x, y, 0.5-z).

## supplementary crystallographic information

### Comment

Schiff base ligands containing the pyridine ring have received considerable attention during the last decades, mainly because their coordinative and electronic properties. For this reason, much effort has been devoted to develop efficient routes for the synthesis of these classes of compounds. In this paper, we report the crystal structure of the title compound, obtained by the reaction of pyridine-2,6-dicarbohydrazide and 4-(dimethylamino)benzaldehyde.

In the title compound (Fig. 1), the bis(4-(dimethylamino)benzylidene)pyridine-2,6-dicarbohydrazide molecule and the water molecule possess crystallographic imposed twofold rotation symmetry. Bond lengths and angles are normal and correspond to those observed in related compounds (Cheng *et al.*, 2007; Cheng & Liu, 2008; Jia, Hu *et al.*, 2006; Jia, Shi *et al.*, 2006). The dihedral angle formed by the benzene ring and the pyridine ring is 17.13 (7)°. In the crystal packing, a two-dimensional supermolecular structure parallel to the *ab* plane is formed by N—H···O and O—H···O intermolecular contacts (Table 1).

### Experimental

To a solution of pyridine-2,6-dicarbohydrazide (3 mmol) in ethanol (30 ml) was added 4-(dimethylamino)benzaldehyde (6 mmol). The mixture was refluxed with stirring for 8 h. An red precipitate was then obtained. Red crystals suitable for X-ray diffraction analysis formed after several weeks on slow evaporation of an ethanol solution at room temperature. Elemental analysis: calculated for C_25_H_29_N_7_O_3_: C 63.14, H 6.15, N 20.62%; found: C 63.28, H 6.22, N 20.49%.

### Refinement

The independent water H atom was located in a difference Fourier map and refined with the O—H bond constrained to 0.85 Å and *U*_iso_(H) = 1.2 *U*_eq_(O). All other H atoms were positioned geometrically and refined using a riding model with C—H = 0.93–0.96 Å, N—H =0.86 Å, and with *U*_iso_(H) = 1.2 *U*_eq_(C, N) or 1.5 *U*_eq_(C) for methyl H atoms

### Crystal data

**Table:**

C_25_H_27_N_7_O_2_·H_2_O	*F*(000) = 1008
*M**_r_* = 475.54	*D*_x_ = 1.332 Mg m^−^^3^
Monoclinic, *C*2/*c*	Mo *K*α radiation, λ = 0.71073 Å
Hall symbol: -C 2yc	Cell parameters from 1533 reflections
*a* = 8.5718 (11) Å	θ = 3.0–24.4°
*b* = 10.2802 (14) Å	µ = 0.09 mm^−^^1^
*c* = 27.112 (3) Å	*T* = 298 K
β = 97.865 (1)°	Block, red
*V* = 2366.7 (5) Å^3^	0.36 × 0.31 × 0.16 mm
*Z* = 4	

### Data collection

**Table:**

Bruker SMART APEX CCD area-detector diffractometer	2076 independent reflections
Radiation source: fine-focus sealed tube	1348 reflections with *I* > 2σ(*I*)
graphite	*R*_int_ = 0.039
phi and ω scans	θ_max_ = 25.0°, θ_min_ = 1.5°
Absorption correction: multi-scan (*SADABS*; Sheldrick, 1996)	*h* = −10→10
*T*_min_ = 0.968, *T*_max_ = 0.986	*k* = −12→12
5774 measured reflections	*l* = −32→17

### Refinement

**Table:**

Refinement on *F*^2^	Primary atom site location: structure-invariant direct methods
Least-squares matrix: full	Secondary atom site location: difference Fourier map
*R*[*F*^2^ > 2σ(*F*^2^)] = 0.062	Hydrogen site location: inferred from neighbouring sites
*wR*(*F*^2^) = 0.204	H atoms treated by a mixture of independent and constrained refinement
*S* = 1.05	*w* = 1/[σ^2^(*F*_o_^2^) + (0.1275*P*)^2^] where *P* = (*F*_o_^2^ + 2*F*_c_^2^)/3
2076 reflections	(Δ/σ)_max_ < 0.001
163 parameters	Δρ_max_ = 0.17 e Å^−^^3^
1 restraint	Δρ_min_ = −0.59 e Å^−^^3^

### Special details

**Table:**

Geometry. All e.s.d.'s (except the e.s.d. in the dihedral angle between two l.s. planes) are estimated using the full covariance matrix. The cell e.s.d.'s are taken into account individually in the estimation of e.s.d.'s in distances, angles and torsion angles; correlations between e.s.d.'s in cell parameters are only used when they are defined by crystal symmetry. An approximate (isotropic) treatment of cell e.s.d.'s is used for estimating e.s.d.'s involving l.s. planes.
Refinement. Refinement of *F*^2^ against ALL reflections. The weighted *R*-factor *wR* and goodness of fit *S* are based on *F*^2^, conventional *R*-factors *R* are based on *F*, with *F* set to zero for negative *F*^2^. The threshold expression of *F*^2^ > σ(*F*^2^) is used only for calculating *R*-factors(gt) *etc*. and is not relevant to the choice of reflections for refinement. *R*-factors based on *F*^2^ are statistically about twice as large as those based on *F*, and *R*- factors based on ALL data will be even larger.

### Fractional atomic coordinates and isotropic or equivalent isotropic displacement parameters (Å^2^)

**Table:**

	*x*	*y*	*z*	*U*_iso_*/*U*_eq_	
N1	0.0000	0.0318 (3)	0.2500	0.0419 (8)	
N2	0.1517 (3)	0.1645 (2)	0.18533 (9)	0.0456 (6)	
H2A	0.0721	0.1938	0.1981	0.068*	
N3	0.2319 (3)	0.2465 (2)	0.15743 (8)	0.0448 (7)	
N4	0.4368 (3)	0.7693 (2)	0.05148 (10)	0.0556 (7)	
O1	0.3055 (3)	−0.0104 (2)	0.17635 (9)	0.0669 (7)	
O2	1.0000	0.3442 (4)	0.2500	0.0927 (13)	
H2	0.934 (4)	0.398 (3)	0.2352 (16)	0.111*	
C1	0.1949 (3)	0.0402 (3)	0.19306 (10)	0.0445 (7)	
C2	0.0935 (3)	−0.0351 (3)	0.22381 (10)	0.0411 (7)	
C3	0.0976 (3)	−0.1702 (3)	0.22298 (11)	0.0488 (8)	
H3	0.1650	−0.2141	0.2046	0.059*	
C4	0.0000	−0.2371 (4)	0.2500	0.0535 (11)	
H4	0.0000	−0.3276	0.2500	0.064*	
C5	0.1730 (3)	0.3604 (3)	0.15200 (10)	0.0462 (7)	
H5	0.0817	0.3786	0.1657	0.055*	
C6	0.2429 (3)	0.4630 (3)	0.12527 (10)	0.0424 (7)	
C7	0.3772 (3)	0.4455 (3)	0.10227 (11)	0.0452 (7)	
H7	0.4255	0.3643	0.1035	0.054*	
C8	0.4387 (3)	0.5447 (3)	0.07816 (11)	0.0474 (8)	
H8	0.5276	0.5292	0.0628	0.057*	
C9	0.3728 (3)	0.6699 (3)	0.07561 (10)	0.0420 (7)	
C10	0.2390 (3)	0.6870 (3)	0.09890 (11)	0.0494 (8)	
H10	0.1917	0.7685	0.0982	0.059*	
C11	0.1755 (3)	0.5864 (3)	0.12276 (11)	0.0489 (8)	
H11	0.0854	0.6010	0.1376	0.059*	
C12	0.5716 (4)	0.7484 (3)	0.02620 (13)	0.0685 (10)	
H12A	0.6567	0.7141	0.0492	0.103*	
H12B	0.6031	0.8295	0.0130	0.103*	
H12C	0.5450	0.6877	−0.0005	0.103*	
C13	0.3639 (4)	0.8964 (3)	0.04783 (13)	0.0639 (9)	
H13A	0.2669	0.8923	0.0255	0.096*	
H13B	0.4335	0.9576	0.0354	0.096*	
H13C	0.3430	0.9235	0.0802	0.096*	

### Atomic displacement parameters (Å^2^)

**Table:**

	*U*^11^	*U*^22^	*U*^33^	*U*^12^	*U*^13^	*U*^23^
N1	0.0486 (18)	0.0423 (18)	0.0350 (17)	0.000	0.0068 (15)	0.000
N2	0.0534 (14)	0.0424 (14)	0.0433 (13)	−0.0004 (11)	0.0155 (11)	0.0021 (11)
N3	0.0518 (14)	0.0457 (15)	0.0376 (13)	−0.0025 (11)	0.0082 (11)	0.0021 (11)
N4	0.0561 (15)	0.0533 (16)	0.0613 (16)	−0.0005 (12)	0.0220 (13)	0.0100 (13)
O1	0.0674 (14)	0.0616 (14)	0.0780 (16)	0.0146 (11)	0.0330 (13)	0.0048 (12)
O2	0.100 (3)	0.081 (3)	0.101 (3)	0.000	0.030 (3)	0.000
C1	0.0497 (16)	0.0449 (17)	0.0392 (16)	0.0056 (13)	0.0068 (13)	−0.0040 (12)
C2	0.0472 (15)	0.0377 (15)	0.0370 (15)	0.0009 (12)	0.0013 (13)	−0.0015 (12)
C3	0.0497 (17)	0.0461 (17)	0.0495 (17)	0.0057 (14)	0.0031 (14)	−0.0055 (14)
C4	0.055 (3)	0.036 (2)	0.066 (3)	0.000	−0.005 (2)	0.000
C5	0.0480 (16)	0.0520 (18)	0.0400 (15)	0.0003 (14)	0.0107 (13)	0.0012 (13)
C6	0.0466 (15)	0.0469 (16)	0.0341 (14)	−0.0018 (13)	0.0072 (12)	0.0000 (12)
C7	0.0443 (15)	0.0449 (16)	0.0472 (16)	0.0026 (13)	0.0091 (13)	−0.0015 (13)
C8	0.0422 (15)	0.0556 (18)	0.0472 (17)	0.0008 (13)	0.0162 (13)	−0.0020 (14)
C9	0.0415 (15)	0.0484 (17)	0.0369 (15)	−0.0024 (13)	0.0080 (12)	0.0008 (13)
C10	0.0512 (17)	0.0456 (17)	0.0533 (18)	0.0064 (14)	0.0134 (14)	0.0008 (14)
C11	0.0503 (16)	0.0510 (18)	0.0496 (17)	0.0024 (14)	0.0220 (14)	0.0010 (14)
C12	0.069 (2)	0.072 (2)	0.071 (2)	−0.0130 (17)	0.0331 (19)	0.0018 (18)
C13	0.073 (2)	0.057 (2)	0.065 (2)	−0.0058 (17)	0.0188 (18)	0.0117 (17)

### Geometric parameters (Å, °)

**Table:**

N1—C2	1.333 (3)	C5—H5	0.9300
N1—C2^i^	1.333 (3)	C6—C11	1.392 (4)
N2—C1	1.339 (3)	C6—C7	1.394 (4)
N2—N3	1.378 (3)	C7—C8	1.356 (4)
N2—H2A	0.8600	C7—H7	0.9300
N3—C5	1.276 (3)	C8—C9	1.404 (4)
N4—C9	1.368 (3)	C8—H8	0.9300
N4—C12	1.438 (4)	C9—C10	1.394 (4)
N4—C13	1.446 (4)	C10—C11	1.371 (4)
O1—C1	1.222 (3)	C10—H10	0.9300
O2—H2	0.85 (3)	C11—H11	0.9300
C1—C2	1.499 (4)	C12—H12A	0.9600
C2—C3	1.390 (4)	C12—H12B	0.9600
C3—C4	1.370 (3)	C12—H12C	0.9600
C3—H3	0.9300	C13—H13A	0.9600
C4—C3^i^	1.370 (3)	C13—H13B	0.9600
C4—H4	0.9300	C13—H13C	0.9600
C5—C6	1.455 (4)		
			
C2—N1—C2^i^	117.8 (3)	C8—C7—H7	119.4
C1—N2—N3	121.5 (2)	C6—C7—H7	119.4
C1—N2—H2A	119.3	C7—C8—C9	122.2 (3)
N3—N2—H2A	119.3	C7—C8—H8	118.9
C5—N3—N2	114.0 (2)	C9—C8—H8	118.9
C9—N4—C12	121.2 (3)	N4—C9—C10	122.1 (3)
C9—N4—C13	120.6 (2)	N4—C9—C8	121.5 (2)
C12—N4—C13	118.0 (2)	C10—C9—C8	116.3 (2)
O1—C1—N2	124.1 (3)	C11—C10—C9	121.6 (3)
O1—C1—C2	121.7 (3)	C11—C10—H10	119.2
N2—C1—C2	114.3 (2)	C9—C10—H10	119.2
N1—C2—C3	122.9 (3)	C10—C11—C6	121.4 (3)
N1—C2—C1	117.8 (2)	C10—C11—H11	119.3
C3—C2—C1	119.3 (2)	C6—C11—H11	119.3
C4—C3—C2	118.3 (3)	N4—C12—H12A	109.5
C4—C3—H3	120.8	N4—C12—H12B	109.5
C2—C3—H3	120.8	H12A—C12—H12B	109.5
C3—C4—C3^i^	119.8 (4)	N4—C12—H12C	109.5
C3—C4—H4	120.1	H12A—C12—H12C	109.5
C3^i^—C4—H4	120.1	H12B—C12—H12C	109.5
N3—C5—C6	122.7 (3)	N4—C13—H13A	109.5
N3—C5—H5	118.7	N4—C13—H13B	109.5
C6—C5—H5	118.7	H13A—C13—H13B	109.5
C11—C6—C7	117.3 (2)	N4—C13—H13C	109.5
C11—C6—C5	119.2 (2)	H13A—C13—H13C	109.5
C7—C6—C5	123.4 (3)	H13B—C13—H13C	109.5
C8—C7—C6	121.2 (3)		

Symmetry codes: (i) −*x*, *y*, −*z*+1/2.

### Hydrogen-bond geometry (Å, °)

**Table:**

*D*—H···*A*	*D*—H	H···*A*	*D*···*A*	*D*—H···*A*
N2—H2A···O2^ii^	0.86	2.23	2.967 (4)	143
O2—H2···O1^iii^	0.85 (3)	2.04 (4)	2.844 (3)	157 (3)

Symmetry codes: (ii) *x*−1, *y*, *z*; (iii) *x*+1/2, *y*+1/2, *z*.
